# The Great Debate at “Melanoma Bridge”, Napoli, December 2nd, 2017

**DOI:** 10.1186/s12967-018-1477-8

**Published:** 2018-04-17

**Authors:** Paolo A. Ascierto, Corrado Caracò, Jeffrey E. Gershenwald, Omid Hamid, Merrick Ross, Ryan J. Sullivan, Igor Puzanov

**Affiliations:** 1 Cancer Immunotherapy and Innovative Therapies, Istituto Nazionale Tumori-IRCCS Fondazione “G. Pascale”, Naples, NA Italy; 2 Division of Surgery of Melanoma and Skin Cancer, Istituto Nazionale Tumori-Fondazione “G. Pascale”, Naples, Italy; 3 Department of Surgical Oncology, The University of Texas MD Anderson Cancer Center, Houston, TX USA; 4 The Angeles Clinic and Research Institute, Cedars Sinai Medical Care Foundation, Los Angeles, CA USA; 5 Center for Melanoma, Massachusetts General Hospital Cancer Center, Harvard Medical School, Boston, MA USA; 6 Department of Medicine, Roswell Park Comprehensive Cancer Center, Buffalo, NY USA

**Keywords:** Melanoma, Lymphadenectomy, Targeted therapy, Immunotherapy, Combination

## Abstract

As part of the 2017 Melanoma Bridge congress (November 30–December 2, 2017, Napoli, Italy), the great debate session featured counterpoint views from leading experts on three contemporary controversial clinical issues in the care of the melanoma patient. These were: (1) whether complete lymph node dissection should be routinely offered to all melanoma patients with sentinel lymph node-positive disease; (2) whether first-line treatment of BRAF-mutated melanoma should consist of BRAF-targeted therapy or immunotherapy with checkpoint inhibitors; and (3) whether combined or sequential administration of treatments should be the preferred option in the management of patients with advanced melanoma. Discussion of these three important issues and audience responses are reported here.

## Introduction

As part of the 2017 Melanoma Bridge congress (November 30–December 2, 2017, Naples, Italy), the great debate session featured counterpoint views from leading experts on three contemporary controversial clinical issues in the care of the melanoma patient. These were: (1) whether complete lymph node dissection should be routinely offered to all melanoma patients with sentinel lymph node-positive disease; (2) whether first-line treatment of BRAF-mutated melanoma should consist of BRAF-targeted therapy or immunotherapy with checkpoint inhibitors; and (3) whether combined or sequential administration of treatments should be the preferred option in the management of patients with advanced melanoma. As the debates were assigned by meeting Chairs, positions taken by each of the Melanoma experts during the debates may not have reflected their respective personal approach. Discussion of these three important issues are summarised below.

## Should completion lymphadenectomy be offered for all patients with sentinel node positive disease?

### Yes (but not always): Merrick Ross

Regional lymph nodes are the most common site of first recurrence in melanoma, with a greater than 50% chance for distant relapse and a 15–50% chance for in-basin failure after a formal therapeutic dissection. To address these poor clinical outcomes, sentinel lymph node (SLN) biopsy was introduced as a strategy to detect and treat regional node disease when microscopic. The goals of SLN biopsy are to provide a minimally invasive low-morbidity approach to nodal staging and to improve disease outcomes for node-positive patients.

Following a positive SLN biopsy in patients with melanoma, complete lymph node dissection (CLND) has been widely accepted as the routine standard of care. The rationale for CLND includes the possibility of a survival benefit and improved regional nodal control compared with delaying lymphadenectomy until nodal disease is clinically apparent. CLND also offers the advantage of improved staging and prognosis. However, the evidence supporting the routine use of CLND has been questioned and attention has been focused on whether the risks of increased surgical morbidity outweigh the potential benefits of CLND.

The average regional recurrence rate after lymphadenectomy alone when treating palpable (macroscopic) nodal disease has been reported to be approximately 20–50% [1]. Risk factors for recurrence were identified as extracapsular extension (ECE), cervical location, and involvement of more than four lymph nodes. In-basin failure after positive SLN biopsy and selective lymphadenectomy has been reported to be much lower, similar to that seen in patients with positive nodes and elective lymph node dissection (9–10%).

A critical question is whether early treatment of lymph node disease improve outcomes. Evidence in favour of this is provided by the Multicenter Selective Lymphadenectomy Trial (MSLT)-I, in which 2001 patients with primary cutaneous melanoma were randomised to undergo wide excision and nodal observation with CLND for nodal recurrence, or wide excision and SLN biopsy with immediate CLND for nodal metastases detected on biopsy [2]. Mean 10-year disease-free survival (DFS) was significantly improved in the biopsy group compared with the observation group in patients with intermediate-thickness melanomas (hazard ratio [HR] for recurrence or metastasis, 0.76; p = 0.01) and thick melanomas (HR, 0.70; p = 0.03). Distant disease-free survival (DDFS) and melanoma-specific survival (MSS) were prolonged with biopsy-based management for patients with nodal metastases from intermediate-thickness melanoma. Still considering this was a post hoc analysis, and therefore results should be confirmed, these data support the proof of concept that treating regional lymph node disease early is clinically beneficial. However, since all of the patients with a positive SLN underwent CLND, it is difficult to assess the fractional benefit of CLND beyond what can be achieved with SLN biopsy alone.

It has been suggested that most, if not all, of the survival benefit observed in the MSLT-I trial can be attributed to the removal of SLNs alone. Non-randomised studies that have compared CLND versus SLN biopsy in SLN-positive patients have suggested that CLND provides no significant survival advantage [3, 4]. However, these studies are retrospective, have imbalances in prognostic factors, significant surgeon and patient bias, and included only short-term follow-up in the group without CLND. As such, they do not support a change in practice away from CLND but do highlight the need for randomised trials with long-term outcomes.

Two such prospective randomised trials comparing observation with nodal basin ultrasound versus CLND in the SLN-positive patient population have been reported. In the MLST-II trial, 1775 patients with SLN metastases were randomised to immediate CLND or nodal observation with ultrasound and were evaluable for per-protocol analysis [5]. Immediate CLND increased the rate of regional disease control and provided prognostic information but did not increase MSS. Similarly, preliminary results of the DeCOG-SLT trial, in which 483 patients with positive SLN biopsy results were randomised to CLND or observation, showed no difference in survival between treatment groups [6]. However, results from these two trials need to be interpreted with caution. Both were underpowered for the at-risk population and favoured patients with a low risk for non-SLN involvement (e.g. in DecOG-SLT, almost 70% of patients were in the < 1 mm SLN tumour burden subgroup). Moreover, the SLN tumour burden subset analyses were retrospective and median follow-up times were of insufficient duration. Also, there is to date no survival analysis to compare the subset of patients in the CLND arm with positive non-SLN involvement versus the subset of patients in the observation arm with nodal basin failure.

Morbidity associated with CLND is high, although it has been shown that symptomatic lymphedema is lower and length of inpatient hospitalization is shorter with immediate CLND versus delayed formal dissection as treatment for nodal basin failure [7]. Only those patients who harbour additional microscopic disease with non-SLN involvement will derive any benefit from CLND. These patients represent approximately 10–20% of SLN-positive patients and studies have indicated that they have a unfavourable prognosis, such that early treatment of nodal disease beyond SLNs may have little impact on MSS [8].

Another argument in favour of CNLD include the ability to provide more accurate and complete staging, with the number of positive nodes and the presence of non-SLN involvement known to affect the predicted risk for distant disease relapse [9]. Positive non-SLN involvement in patients undergoing CLND is also a strong independent predictor of disease-specific survival in patients with melanoma [10] and, as such, CLND represents an excellent staging tool. Accurate staging may be critical when assessing the risk: benefit ratio of systemic adjuvant therapy, especially given the high toxicity profiles and costs of currently approved therapies.

Improved regional disease control and reduced post-dissection morbidity for patients with non-SLN involvement remains an important treatment goal. Durable regional nodal basin disease control is improved when regional lymphadenectomy is performed to treat microscopic nodal disease and surgical morbidity of the regional dissection is lower when treating SLN metastases compared with treating patients who have palpable nodal disease [11, 12].

In conclusion, there is admittedly no direct evidence that CLND provides a survival benefit beyond what is achieved by removing the involved SLNs and, therefore, the SLN biopsy may be therapeutic for patients with SLN disease only (representing around 80% of SLN-positive patients). Moreover, prognosis in patients with non-SLN involvement is particularly unfavourable and these patients may not benefit from CLND. However, despite this, up to one-fifth of patients have additional lymph nodes involved identified by routine histology and this incidence of non-SLN involvement may actually be under-estimated. As such, a significant proportion of patients have the possibility to benefit from CLND. Because of this, a selective decision on whether to perform CLND based on predicted risk of non-SLN involvement is a rational approach.

### No (but maybe sometimes): Jeffrey E. Gershenwald

Assessment of clinically negative regional lymph nodes is useful for staging and prognosis as well as offering a possible survival benefit and improved regional node basin control. SLN status is the most powerful predictor of survival in stage I–II melanoma and the single most important prognostic factor in patients with clinically node-negative melanoma [2]. Improved MSS for patients with nodal metastases with intermediate-thickness melanoma who undergo wide excision with SLN biopsy versus wide excision and nodal observation supports the hypothesis that clinically-occult metastases become clinically evident [2].

Proponents of CLND argue that it should be performed for SLN-positive patients on the basis that non-SLN metastases can be identified and removed and that it is of prognostic significance and allows complete staging, which includes the possibility of upstaging some patients with a consequent influence on clinical decision-making. Moreover, CLND may reduce in-basin failure and loss of regional control, and may also have a positive effect on survival. However, there are several challenges to this strategy. Only 9–20% of patients have tumour-involved non-SLNs and so only a fraction of SLN-positive patients can derive any benefit from CLND. Moreover, there are other predictors of non-SLN involvement and adverse survival that can be used without definitive need for CLND, since patients with a high risk of non-SLN tumour involvement are also considered to be at high risk of distant disease and death from melanoma.

Importantly, there is no evidence that early treatment of regional SLN disease improves survival. In the DeCOG trial, there were no overall significant differences in 5-year recurrence-free survival (RFS), distant metastasis-free survival (DMFS), or MSS between patients randomised to CLND or observation at a median follow-up of 35 months [6]. Similarly, in the larger MLST II study, early CLND did not increase melanoma-specific survival at the reported median follow-up of 43 months [7]. DFS was slightly higher in the CLND group, which appeared to result from a reduction in the rate of nodal recurrence after CLND and corresponded to an increase in the disease control rate (DCR) in the regional nodes at 3 years. Recurrence in the nodal basin, as a sole site of recurrence, was observed in 7.7% in the observation group compared with 1.3% in the dissection group; this cohort of patients was distinct from those who had nodal recurrence that occurred with either locoregional disease or systemic disease. There was also no significant difference in distant metastasis-free survival (DMFS) between the CLND and observation groups. A subgroup analysis, including an exploratory analysis based on SLN tumour burden, did not reveal any subgroups that derived a significant MSS benefit from CLND.

CLND is also proposed as being important for more accurate staging. While it was associated with improved staging and regional control, it should be considered what proportion of patients, if any, would be upstaged and receive adjuvant therapy, or perhaps be offered a different adjuvant therapy regimen, *solely* on the basis of the pathological results of the CLND (i.e., presence or absence of non-SLN disease). In the MD Anderson Cancer Center experience, the long-term incidence of additional positive non-SLN involvement among patients who underwent CLND after a positive SLN was 14%, which is similar to that reported in other studies [11]. In patients treated from 1996 onwards, the incidence was only 10.5% [11].

Other factors can be used to predict non-SLN involvement. In our study of patients with clinically node-negative melanoma who underwent SLN biopsy and had positive SLNs, SLN microscopic tumour burden, tumour thickness > 2 mm and fewer SLNs harvested (i.e., 1 versus 2 versus 3 or more) were predictive of increased non-SLN involvement [11].

In a multivariate analysis of prognostic factors in patients with stage III melanoma and nodal micrometastases, the number of tumour-containing lymph nodes, primary tumour thickness, patient age, ulceration, and anatomic site of the primary lesion all independently predicted survival [13]. Primary tumour mitotic rate was the second-most powerful predictor of survival after the number of tumour-containing nodes. Higher-risk patients can likely be identified by contemporary analytic approaches based on modelling various prognostic factors without the need for CLND.

In conclusion, trial data published to date are unequivocal that there has been no survival benefit associated with CLND [6, 7]. CLND has been associated with significant morbidity versus observation. Active surveillance appears to be safe in this era of improved imaging techniques, frequent use of nodal ultrasound as a component of ‘active surveillance’, and effective adjuvant therapy. Clinical decision-making related to delayed CLND can be performed during active surveillance at the time of initial recurrence, if any. Since isolated regional recurrence that may warrant consideration of ‘delayed’ CLND may also be detected when the overall disease burden is low compared to historical patterns, the increased morbidity associated with CLND reported in legacy studies when clinical disease is present may no longer be as relevant. Clinically relevant upstaging based on CLND is limited. On this basis, CLND should not be routinely offered to patients with a positive SLN biopsy.

**Fig. 1:**
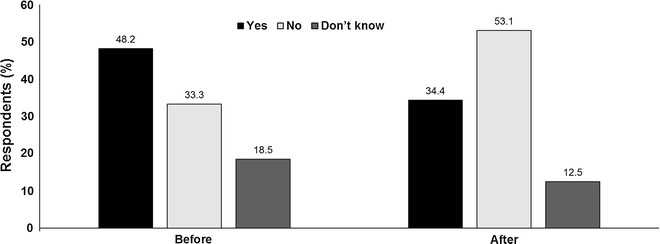
Should completion lymphadenectomy be offered for all patients with sentinel node positive disease? Audience response

*Audience vote* Before the debate, almost half of delegates (48%) were in favour of routine CLND for patients with positive SLN biopsy. However, this declined to just over a third (34%) while the percentage against immediate CLND increased from one-third to 53%. The proportion of patients who were unsure also decreased (Fig. 1).

### Key points

- Interim published results from two randomised clinical trials [6, 7] have thus far demonstrated no survival benefit associated with CLND for patients with a positive SLN, although CLND was associated with improved staging and regional node control.
- Active surveillance for patients who do not undergo CLND following a positive SLN should include nodal ultrasound as component of the follow-up strategy.
- Despite the results reported to date, the survival benefit associated with CLND, if any, remains controversial. Some proponents of CLND argue that the randomised clinical trials included only a fraction of patients with significant SLN tumour burden (i.e., at higher risk of non-SLN tumour involvement), and that non-SLN tumour involvement is likely underestimated by routine histology.
- CLND should be discussed with patients as part of a dialogue of the risks, benefits, and alternatives of the procedure, including their overall risk of harbouring tumour-involved non-SLNs, and the impact of CLND on staging, regional control, and survival.

## Should immunotherapy or targeted therapy be the first-line treatment choice for BRAF-mutated melanoma?

### In favour of targeted therapy first: Ryan Sullivan

Novel agents that are directed against immune checkpoint molecules or mutated BRAF are therapeutic options for patients with BRAF-mutant melanoma. However, the most effective first-line treatment and the optimal sequencing of these agents is not well characterised. For some patient groups, the choice of whether first-line treatment should be immunotherapy or BRAF therapy is clear. For example, patients with high lactate dehydrogenase (LDH) (i.e., > 2× ULN) should receive combined ipilimumab and nivolumab as this offers the best long-term survival outcomes in this population [14], while patients with rapidly progressive disease that is immediately life-threatening (e.g. impending organ failure, viscus obstruction, etc.) and/or associated with brain metastases requiring high-dose steroids should be treated with combined BRAF/MEK inhibitor therapy. However, the choice of first-line therapy for all other patients is less obvious. While MAPK inhibitors can provide early and high rates of response, immunotherapy typically offers more durable responses and potentially longer-term disease control, although with a slower onset of effect. Indeed, it has been suggested that, although targeted therapy may be preferable in patients with high tumour burden and symptomatic disease who require rapid improvement, first-line treatment with immune checkpoint inhibitors may be preferable in patients who do not require immediate control of symptoms.

Some retrospective data have suggested that BRAF-targeted therapy is useful after ipilimumab, while ipilimumab is not as effective after BRAF-targeted therapy. In one study, prior treatment with ipilimumab (or high-dose interleukin-2) did not appear to negatively influence subsequent response to BRAF inhibitor therapy [15]. However, outcomes with ipilimumab following BRAF inhibitor discontinuation were poor. In another analysis, overall survival [OS] improved in patients who were treated with ipilimumab before BRAF inhibitor compared with those treated with BRAF inhibitor followed by ipilimumab (median OS 14.5 versus 9.7 months) [16].

More recently, in a retrospective assessment of 114 patients, BRAF inhibitor therapy after progression on anti-PD-1 therapy was not particularly effective. Specifically, as might be expected patients who progressed on anti-PD-1 had worse progression-free survival (PFS) with subsequent BRAF inhibitor therapy than those treated with BRAF inhibitor therapy prior to anti-PD-1 [17]. Similarly, patients who previously progressed on a BRAF inhibitor had inferior outcomes after starting anti-PD-1 compared with those without prior BRAF inhibitor treatment. Yet, patients who started BRAF inhibitor therapy first followed by anti-PD-1 had better outcomes compared to patients who started anti-PD-1 first followed by BRAF inhibitor therapy, despite baseline and features that might predict better outcomes (less likely to have brain metastases at baseline, more likely to have a normal LDH at baseline, more likely to receive combined immune checkpoint inhibitor therapy).

Additional information to support frontline BRAF-targeted therapy comes from data showing that the cohort of patients most likely to achieve long-term benefit from BRAF/MEK inhibition has been identified as those with normal serum LDH and less than three disease sites [18]. In a 3-year pooled analysis of factors associated with clinical outcomes across phase III trials of dabrafenib and trametinib combination therapy, baseline LDH level and number of disease sites were strongly associated with PFS and OS [19]. Baseline sum of lesion diameters was also identified as a predictor for disease progression. Moreover, the use of BRAF/MEK inhibitors (dabrafenib and trametinib) as adjuvant treatment for patients with completely resected, stage III BRAF-mutated melanoma was associated with improved PFS and OS without any new safety signals [20]. These data suggest that patients with low disease burden can achieve durable responses to BRAF/MEK inhibition and may represent the most appropriate group to treat with first-line targeted therapy.

In summary, retrospective data supports upfront treatment with BRAF targeted therapy before immunotherapy, perhaps due to shared mechanisms of resistance seen after frontline anti-PD-1 than frontline BRAF targeted therapy. Moreover, BRAF/MEK inhibitor therapy is associated with durable benefit in a subset of patients, i.e., those with lower disease volume (normal LDH, < 3 sites of disease). However, ultimately the decision about whether to treat with targeted therapy or immunotherapy first requires a randomised controlled trial.

### In favour of immunotherapy first: Omid Hamid

Immunotherapy with checkpoint inhibitor therapy provides durable long-term survival for patients with melanoma. In a pooled analysis of ipilimumab trials with 1861 patients, 3-year survival rate was 22% and median OS was 11.4 months [21]. In a trial to compare ipilimumab 10 mg/kg with ipilimumab 3 mg/kg in patients with advanced melanoma, median OS was 15.7 months compared with 11.5 months [22]. Better outcomes still have been achieved with combined regimens. In a pooled 3-year analysis of data from phase II–III trials of nivolumab combined with ipilimumab in advanced melanoma, OS rate was 73.3% at 1-year, 64.1% at 2 years and 57.9% at 3 years; median OS was not reached at a median follow-up of 37.5 months [23]. These studies all indicate the excellent long-term survival outcomes that can be achieved with checkpoint inhibitors, especially in combination.

However, resistance to immunotherapy, both innate and acquired, remains a problem. Various mechanisms of primary resistance have been proposed, including the loss of major histocompatibility complex (MHC), an increase of the number of regulatory cells into the tumour microenvironment, and an increase of the production of immunosuppressive cytokines. Recently, tumours with innate resistance have been found to display a transcriptional signature, the innate anti-PD-1 resistance (IPRES) signature, that reflects concurrent activation of processes such as extracellular-matrix remodelling, hypoxia, angiogenesis and wound-healing [24]. This may indicate a link between cancer mesenchymal state and an immune-suppressive tumour microenvironment. Importantly, MAPK-pathway targeted therapy induces similar signatures in melanoma, suggesting that a non-genomic form of MAPK inhibitor resistance mediates cross-resistance to anti-PD-1 therapy. Five of 26 signatures that defined IPRES were induced early with MAPK targeted therapy. Thus, resistance to targeted therapy in melanoma is associated with the acquisition of highly recurrent non-genomic alterations as well as changes in the immune tumour microenvironment that may result in cross-resistance to anti-PD-1/PD-L1 therapy. Shared mechanisms of resistance with immunotherapy and MAPK inhibitors may be an important consideration in choice of first-line treatment.

Even more impressive outcomes are now being observed through the use of various combination strategies, including combining existing checkpoint inhibitors with other novel immunotherapeutic approaches. One such example of this is the combination of anti-PD-1 agents with epacadostat, a potent and specific oral inhibitor of the IDO1 enzyme. In an open-label, phase I/II study (ECHO-202/KEYNOTE-037) in multiple tumour types, epacadostat plus pembrolizumab showed promising antitumor activity in patients with advanced melanoma [25]. In 63 melanoma patients, overall response rate (ORR) was 56% (complete responses [CR] 14%) and DCR was 71%. Median PFS was 12.4- and 18-month PFS was 49%. Epacadostat plus pembrolizumab had a favourable safety profile with treatment-related grade 3/4 adverse events reported in 20% of patients. Another potential combination with anti-PD-1 therapy is anti-lymphocyte-activation gene (LAG)-3, an immune checkpoint receptor that regulates T cell function. In an ongoing expansion study of 48 heavily pre-treated patients with advanced melanoma refractory to or relapsed on anti-PD-1/PDL-1 therapy, an ORR of 12.5% was observed with the anti-LAG-3 agent BMS-986016 (relatlimab) in combination with nivolumab [26]. Patients with LAG-3 tumour expression ≥ 1% had a nearly threefold improvement in ORR compared to patients with < 1% LAG-3 expression (20% versus 7.1%). The safety profile of the combination was similar to nivolumab monotherapy.

Immunotherapy has also shown success in patients with brain metastases. In the phase II CheckMate 204 study in 75 patients, nivolumab plus ipilimumab had a high intracranial ORR of 55% (21% CR) at a median follow-up of 9.2 months [27]. Six-month PFS was 67% with median PFS not reached. The safety profile of the combination was consistent with that in patents without brain metastases, with no unexpected CNS safety signals.

The use of checkpoint inhibitors as adjuvant therapy is also now a focus of attention. In the phase III CheckMate 238 trial, nivolumab 3 mg/kg was compared with ipilimumab 10 mg/kg in 906 patients after complete resection of stage IIIB–IV melanoma [28]. Recurrence-free survival (RFS) at 1 year was 70.5% (95% CI 66.1–74.5) in the nivolumab group compared with 60.8% (95% CI 56.0–65.2) in the ipilimumab group (HR for disease recurrence or death, 0.65; 97.56% CI 0.51–0.83; p < 0.001). Nivolumab was better tolerated than ipilimumab, with fewer grade 3–4 treatment-related adverse events (TRAEs) (14.4% versus 45.9%) or treatment-related discontinuations (9.7% versus 42.6%).

In conclusion, first-line immunotherapy is associated with prolonged long-term survival compared with BRAF/MEK inhibition. Overcoming primary resistance is an important goal and new combinations of checkpoint inhibitors with other immunotherapies offers the potential for further improvements. Better understanding of predictive/prognostic factors and the identification of clinically useful biomarkers will also help to improve treatment outcomes.

**Fig. 2:**
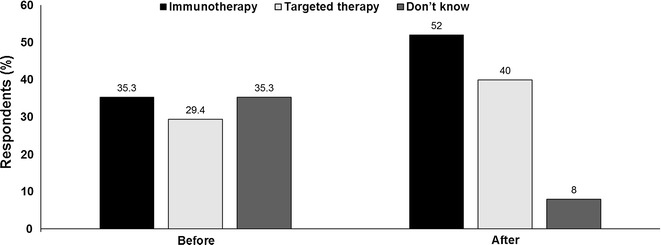
Should immunotherapy or targeted therapy be the first-line treatment choice for BRAF-mutated melanoma? Audience response

*Audience vote* The proportions of the audience in favour of either immunotherapy, targeted therapy or who were unsure as to first-line treatment choice for BRAF-mutated melanoma were fairly well balanced before the debate. However, post-debate, the number of don’t knows declined from 35% to just 8%, while proponents for both immunotherapy and targeted therapy as first-line treatment had increased, with immunotherapy the most popular option (52%) (Fig. 2).

### Key points

- Prospective and randomised data to help guide frontline treatment selection of BRAF-targeted therapy or immune therapy in patients with BRAF-mutant melanoma is lacking.
- Retrospective data and post hoc analyses of prospective trial data supports the selection of BRAF-targeted therapy, particularly in patients with a normal LDH and limited number of disease sites (< 3).
- Cross-trial comparisons of the major randomised trials (COMBI-v, COMBI-d, CoBRIM, KN006, CheckMate-067) suggests that landmark PFS at 3 years and beyond is better with anti-PD-1 therapy either as a single agent or in combination with ipilimumab.
- Adjuvant data from COMBI-ad and CheckMate-238 supports the use of either dabrafenib plus trametinib or nivolumab in patients with resected, high-risk BRAF-mutated melanoma.

## Combination or sequential treatment administration for patients with advanced melanoma?

### In favour of combination administration: Igor Puzanov

Combined immunotherapy approaches have existed for over 30 years, since the administration of autologous lymphokine-activated killer cells plus interleukin (IL)-2 reported in 1985 [29], and today represents the next step in the incorporation of immuno-based therapy into cancer care. The combination of anti-CTLA-4 and anti-PD-1 therapies has been shown to provide superior PFS and ORR in comparison to single-agent ipilimumab in patients with melanoma. In the phase III Checkmate 067 trial, 945 treatment-naïve patients were randomised to nivolumab alone, nivolumab plus ipilimumab, or ipilimumab alone [30]. At a minimum follow-up of 28 months, median OS was not reached in the combination or nivolumab alone groups, and was 20 months for ipilimumab (HR: combination versus ipilimumab, 0.55; p < 0.0001; nivolumab versus ipilimumab, 0.63; p < 0.0001). Two-year OS rates were 64% for the combination, 59% for nivolumab alone and 45% for ipilimumab alone. However, the study was not designed to compare the combination of ipilimumab plus nivolumab with nivolumab monotherapy. TRAEs leading to discontinuation were higher in the combined group (39.6% versus 11.5% with nivolumab alone and 16.1% with ipilimumab alone); however, the ORR was 70.7% for patients who discontinued combination therapy due to TRAEs, with median OS not reached.

In addition to combinations of approved checkpoint inhibitors, a large number of combinations involving the addition of novel immunotherapeutic agents to anti-PD-1/anti-CTLA-4 treatment are being actively explored. Pegylated IL-2 (NKTR-214) is a CD122-biased immune-stimulatory cytokine that selectively binds to the IL-2 receptor-β/γ. Biased signalling preferentially activates and expands T effector cells and natural killer (NK) cells over T regulatory cells and increases proliferation of tumour-infiltrating lymphocytes (TILs) and PD-1 expression on T effector cells in the tumour microenvironment. In the ongoing phase 1/2 PIVOT-02 study of NKTR-214 plus nivolumab in patients with selected solid tumours, ORR was 64% by RECIST (71% by immune-related RECIST) and DCR was 91% in 11 treatment-naïve patients with advanced melanoma [31]. The combination was well tolerated with no study discontinuations due to TRAEs and no treatment-related deaths. NKTR-214 did not increase the risk for immune-related TRAEs associated with nivolumab.

Another novel approach involves the use of toll-like receptor (TLR) 9 agonists. TLR9 induces interferon (IFN)-α and the maturation of antigen-presenting cells, resulting in TIL proliferation. The combination of dendritic cell activation with checkpoint inhibition may offer benefits to immunotherapy-refractory patients. In a phase I/II trial, the TLR9 agonist IMO-2125 was administered intratumourally to patients with PD-(L)1-refractory melanoma in combination with ipilimumab (n = 18) or pembrolizumab (n = 4) [32]. No dose-limiting toxicities were reported. Clinical benefit was observed, with biopsies showing maturation of the mDC1 subset (CD1c+, CD303−), upregulation of PD-L1 by malignant cells, and an IFN-α response gene signature. Moreover, biopsies of uninjected tumours showed evidence of an abscopal effect, with expression of CD56+ and Ki67+ effector CD8+ T cells in responding patients. Several other combinations are also being investigated and have shown promise, including ipilimumab with the oncolytic virus, talimogene laherparepvec (T-VEC), as well as anti-PD-1 agents in combination with selective histone deacetylase (HDAC) inhibitors (entinostat), anti-LAG-3 agents, glucocorticoid-induced tumor necrosis factor receptor (GITR) agonists and OX-40 inhibitors.

One critical issue is the development of biomarkers to allow a more precise choice of immunotherapy. Total tumour mutational burden has been indicated as a potential biomarker and has been shown to correlate with patient response to checkpoint inhibition. Using a RNA-sequencing profiling approach in a cohort of 167 patients with various solid tumours (melanoma, non-small-cell lung cancer [NSCLC], renal cell cancer, bladder cancer, or head and neck cancer), PD-L1 over-expression was more common in inflamed tumours than non-inflamed tumours (19% versus 9%) (ref required [33]. However, the rate of high mutational burden was slightly higher in non-inflamed tumours (16%) than in inflamed tumours (14%). This suggests that mutational load as a single biomarker is less than optimal given our understanding of the importance of TILs and the various facets of T cell activation or suppression. A rational approach to selection of combination immunotherapy is necessary, as can be achieved through an evaluation of the intersection of high mutational burden and PD-L1 over-expression with a set of known targets of immunomodulatory agents. Such a precision immunotherapy based approach offers the potential for better choice of combination immunotherapy for patients.

Combinations strategies are the next step in immunotherapy and, although the obstacles are many and high and include the increasing costs of treatment, patient selection, low patient participation in clinical trials and the need to evaluate multiple combinations, the potential rewards may be even higher.

### In favour of sequencing: Paolo Ascierto

The combination of different immunotherapies has clearly been shown to offer benefits compared with single-agent therapy. In the CheckMate 067 trial of ipilimumab plus nivolumab, PFS at longer-term follow-up was consistent with the primary 9-month analysis and both the nivolumab and the nivolumab plus ipilimumab groups continued to show significant improvements in PFS versus ipilimumab alone [30]. The reduction in the risk of progression or death was 46% for the nivolumab versus ipilimumab group and 58% for the combination versus ipilimumab group, with an apparent separation between the curves appearing as early as 3 months. The relative reduction in the risk of death for the combination versus nivolumab was 15% and separation between the survival curves for the combination arm compared with the two monotherapy arms was maintained over time; median OS for nivolumab plus ipilimumab was still not reached, and was 38 months for nivolumab alone. Looking at the 3-year OS rate, 58% of treated patients were still alive with the combination versus 52% of nivolumab-treated patients. The difference was only 6%. Considering that in the nivolumab alone arm, 46% of patients received subsequent treatment (most frequently ipilimumab) compared to 32% in the combination arm, this difference could be interpreted as the difference between two approaches: the combination of nivolumab/ipilimumab versus the sequencing of nivolumab and ipilimumab. Similarly, data from the CheckMate 069 trial suggest that the combination of first-line nivolumab plus ipilimumab might lead to improved outcomes compared with first-line ipilimumab alone; at a median follow-up of 24.5 months, 2-year OS was 63.8% (95% CI 53.3–72.6) with nivolumab plus ipilimumab versus 53.6% (95% CI 38.1–66.8) with ipilimumab alone, with median OS not reached in either group [34]. The difference in the 2-year OS rate was only about 10%, much less that the difference in the 2-year PFS rate (51% versus 12%). The main reason for the ‘recovery’ in OS with ipilimumab monotherapy was that 57% of patients who progressed were treated with nivolumab after progression; again, this compares sequencing of ipilimumab-nivolumab versus the combination approach.

Potential combination strategies for the treatment of cancer also represent potential sequencing strategies and sequential administration of nivolumab followed by ipilimumab, or the reverse sequence, was assessed in the CheckMate 064 trial [35]. The proportion of patients with a response after 25 weeks was higher with nivolumab followed by ipilimumab than with the reverse sequence (41% versus 20%). Disease progression was reported in 38% of patients in the nivolumab followed by ipilimumab group compared with 60% in the ipilimumab followed by nivolumab group. After a median follow-up of almost 20 months, median OS was not reached in the nivolumab followed by ipilimumab group whereas median OS was 16.9 months in the ipilimumab followed by nivolumab group over a median follow-up of 14.7 months. More patients in the nivolumab followed by ipilimumab group achieved 1-year OS than in the reverse sequence group (76% [95% CI 64–85] versus 54% [95% CI 42–65). TRAEs of grade ≥ 3–5 were similar in both treatment arms. These data suggest nivolumab followed by ipilimumab appears to be a more clinically beneficial option compared with the reverse sequence, although associated with slightly increased toxicity. However, the high toxicity rate observed in this study was probably due to the insufficient interval between the two treatments which resulted in exposure to both drugs at the same time (similar to combination therapy) when switching treatment. In fact, in another trial (CA209-066), patients who progressed on nivolumab were treated with ipilimumab with no additional toxicity to that known from the previous experience. In this study, the median wash-out before starting ipilimumab was 4 weeks, longer than that in the CheckMate 064 study. Moreover, the median OS of patients who received ipilimumab subsequent to nivolumab was 9.0 months from the start of treatment with ipilimumab [36]. One and 2-year survival rates were comparable to those seen with ipilimumab in other trials.

If we also consider that higher dosages of ipilimumab achieved a better OS than the classical 3 mg/kg dose [22], we could consider a possible sequencing schedule for the future of nivolumab with ipilimumab 10 mg/kg given 4 weeks before nivolumab treatment. This sequence might achieve a slightly improved OS than the sequence retrospectively evaluated in the previous clinical trials.

Targeted therapy with immunotherapy is also a rational combination for advanced BRAFV600 mutant melanoma. Targeted therapy offers a rapid and clinically significant tumour response while immunotherapy provides more durable responses. Combining the two approaches offers the possibility of both a rapid and durable tumour response and prolonged survival. Moreover, BRAF inhibitors have been shown to have an effect on the immune system, BRAF inhibition is associated with increased melanoma antigen expression and increased CD8+ T-cell infiltrate in tumours of patients with metastatic melanoma [37]. These data suggest that treatment with BRAF inhibition facilitates a more favourable tumour microenvironment, providing support for the idea of potential synergy of BRAF-targeted therapy and immunotherapy.

Clinical trials to assess the combination of targeted BRAF/MEK inhibitors with immunotherapy are being conducted. In a phase I study (NCT02027961), the PD-L1 inhibitor durvalumab 3 or 10 mg/kg every 2 weeks in combination with a BRAF inhibitor (dabrafenib) and MEK inhibitor (trametinib) had a manageable safety profile and evidence of clinical activity in patients with stage IIIc/IV melanoma [38]. Patients with a BRAF mutation treated with a combination of BRAF and MEK inhibition exhibited the greatest immune activation as well as the greatest clinical activity. Another ongoing phase I study is KEYNOTE-022, in which pembrolizumab combined with dabrafenib and trametinib is being assessed in patients with advanced BRAF-mutated melanoma. In preliminary data, 15 patients were treated with pembrolizumab at 2 mg/kg every 3 weeks and dabrafenib 150 mg twice daily with trametinib 2 mg daily [39]. Dose-limiting toxicities were reported in three patients who discontinued treatment (grade 4 neutropenia, grade 4 increased alanine aminotransferase (AST), and grade 3 increased aspartate transaminase, ALT and gamma-glutamyltransferase). All events resolved and no treatment-related deaths were observed. No late or unexpected toxicities were reported with longer follow-up. The confirmed ORR was 67% (CR 13%). Seven of 11 patients with a response have not progressed with median follow up of around 20 months. In a further study, the triplet combination of atezolizumab plus cobimetinib plus vemurafenib had a manageable safety profile in patients with BRAF V600-mutated metastatic melanoma. Adverse events with the triple combination were similar to those observed with atezolizumab plus vemurafenib. The triple combination showed promising antitumour activity with a non-confirmed response rate of 83% (95% CI 64.2–94.2) [40].

However, the question remains whether the triplet combination with its additional toxicity, even if well managed, is really needed or whether the same results in terms of OS can be achieved with sequential targeted therapy and immunotherapy. The ongoing phase III study will hopefully help answer this question. In the future, it would also be interesting evaluate the combination/sequencing approach with tyrosine kinase inhibitors and anti-PD-1/PD-L1 therapy in patients with aggressive disease, such as those with elevated LDH, high tumour burden and brain metastases.

Ongoing studies will further help identify the optimal sequential approach. These include the SECOMBIT trial, a three arm non-comparative randomised study, which will assess combination immunotherapy (ipilimumab plus nivolumab) followed by combination targeted therapy (encorafenib plus binimetinib) or the reverse sequence in patients with BRAF-mutated melanoma, and a third arm with an induction phase (8 weeks) with combination targeted therapy, switching to combination immunotherapy at best response, and switching back to the targeted combination at progression (NCT02631447).

Finally, sequential immunotherapy with chemotherapy may also offer promise. In a phase II trial to evaluate ipilimumab in combination with chemotherapy (carboplatin/paclitaxel) in lung cancer, phased ipilimumab, but not concurrent ipilimumab, improved immune-related PFS versus placebo (HR = 0.64; p = 0.03) in patients with extensive-disease-small-cell lung cancer [41]. Phased ipilimumab, concurrent ipilimumab and placebo were associated with median immune-related PFS of 6.4, 5.7 and 5.3 months, respectively. In patients with chemotherapy-naive NSCLC, phased ipilimumab also improved PFS according to modified WHO criteria (HR, 0.69; p = 0.02) [42]. Phased ipilimumab, concurrent ipilimumab, and control treatments were associated with a median immune-related PFS of 5.7, 5.5, and 4.6 months, respectively.

While combination approaches clearly represent the next step forward in melanoma management, the question of whether combination or sequential administration is preferable remains largely unanswered. Although clinical trials are required to definitively address this question, the option of sequencing treatments needs to be considered.

**Fig. 3:**
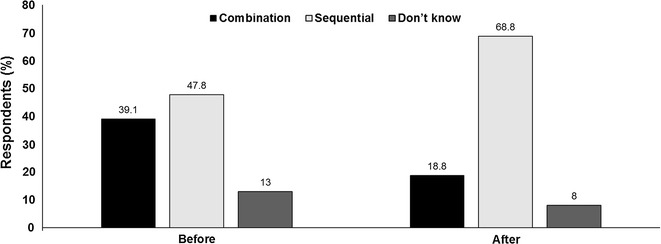
Combination or sequential treatment administration for patients with advanced melanoma? Audience response

*Audience vote* Sequential administration was the audience’s preferred choice before the debate, with 48% in favour versus 39% who favoured combination treatment. After the discussion, sequential treatment proved even more popular, with 69% favouring this option while the percentage in favour of combination declined to 19% (Fig. 3).

### Key points

- There is a lack of prospective and randomised data to help guide the choice of sequential versus combination therapy treatment selection in patients with unresectable or metastatic melanoma.
- Data from the CheckMate-067 trial were not planned to compare ipilimumab plus nivolumab versus nivolumab alone so we can only can guess if the 5% OS difference is statistically significant.
- In the BRAF-mutated population, an ongoing clinical trial with the triplet combination (BRAF/MEK inhibitors plus anti-PD-1/PD-L1) will provide additional information about the roles of combination or sequential therapy.
- Novel immunotherapy combinations are being pursued to provide less toxic options for patients.
